# Ferroptosis-immune crosstalk in cervical cancer: mechanisms and therapeutic implications

**DOI:** 10.3389/fimmu.2025.1657905

**Published:** 2025-08-15

**Authors:** Lili Li, Yunfeng Bo, Dan Xue, Lijuan Qin

**Affiliations:** ^1^ Department of Radiotherapy, Shanxi Province Cancer Hospital/Shanxi Hospital Affiliated to Cancer Hospital, Chinese Academy of Medical Sciences/Cancer Hospital Affiliated to Shanxi Medical University, Taiyuan, ShanXi, China; ^2^ Department of Pathology, Shanxi Province Cancer Hospital/Shanxi Hospital Affiliated to Cancer Hospital, Chinese Academy of Medical Sciences/Cancer Hospital Affiliated to Shanxi Medical University, Taiyuan, Shanxi, China; ^3^ Shanxi Cancer Hospital/Shanxi Hospital Affiliated to Cancer Hospital, Chinese Academy of Medical Sciences/Cancer Hospital Affiliated to Shanxi Medical University, No.3 Zhigong Xincun Street, Xinghualing District, Taiyuan, Shanxi, China

**Keywords:** cervical cancer, ferroptosis, immune evasion, tumor microenvironment, immunotherapy

## Abstract

Cervical cancer remains a leading cause of cancer-related mortality in women worldwide, particularly in regions with limited access to screening and vaccination. While immunotherapy has shown promise in treating advanced cervical cancer, immune evasion mechanisms within the tumor microenvironment continue to limit therapeutic efficacy. Ferroptosis, a form of iron-dependent regulated cell death characterized by lipid peroxidation, has recently been recognized as a crucial regulator of tumor progression and immune modulation. Emerging evidence suggests that ferroptosis interacts with immune signaling pathways, contributing to immune suppression, antigen presentation defects, and the remodeling of the tumor immune microenvironment in cervical cancer. This review highlights the current understanding of ferroptosis-related mechanisms underlying immune evasion in cervical cancer, including alterations in ferroptosis regulators, redox imbalance, and ferroptosis-induced release of immunomodulatory molecules. We further explore how targeting ferroptosis may enhance anti-tumor immunity and overcome resistance to immunotherapy. Finally, we discuss recent advances in ferroptosis-based therapeutic strategies and identify future directions for integrating ferroptosis modulation into cervical cancer treatment.

## 1 Introduction

Cervical cancer remains one of the most prevalent malignancies affecting women worldwide, with over 600,000 new cases and 340,000 deaths reported in 2022 from the World Health Organization (WHO), predominantly in low- and middle-income countries where access to HPV vaccination and screening remains limited. Despite significant progress in prevention and treatment, especially through the implementation of immunotherapeutic strategies such as PD-1/PD-L1 checkpoint blockade, durable responses are seen only in a subset of patients (1, 2). A major contributing factor to this limited efficacy is the ability of cervical cancer cells, especially those driven by high-risk human papillomavirus (HPV) strains such as HPV16 and HPV18, to escape immune surveillance through multiple mechanisms, including impaired antigen presentation, suppression of cytotoxic T cell infiltration, and recruitment of immunosuppressive myeloid populations (3, 4). The complexity of immune evasion in the cervical tumor microenvironment (TME) underscores the urgent need to identify novel regulatory pathways that modulate immune activity and determine therapeutic responsiveness (5, 6).

Ferroptosis, first defined in 2012 as an iron-dependent, lipid peroxidation-driven form of regulated cell death, has since emerged as a critical process in cancer biology with unique implications for immunogenicity (7, 8). Unlike apoptosis or necroptosis, ferroptosis is characterized by the accumulation of lethal lipid reactive oxygen species (ROS) and iron overload, and is tightly controlled by metabolic regulators such as glutathione peroxidase 4 (GPX4), SLC7A11, and ferroptosis suppressor protein 1 (FSP1) (9). Intriguingly, recent studies reveal that ferroptotic tumor cells can actively shape the immune landscape through the release of damage-associated molecular patterns (DAMPs), modulation of cytokine signaling, and alteration of antigen presentation machinery. In breast and pancreatic cancers, ferroptosis has been shown to either promote anti-tumor immunity or exacerbate immune escape, depending on the context. However, the role of ferroptosis in HPV-driven cancers, including cervical cancer, remains underexplored—particularly in how it interfaces with immune escape pathways.

Recent transcriptomic and single-cell analyses of cervical cancer tissues have begun to uncover ferroptosis-related gene signatures correlated with immune exclusion and poor prognosis, suggesting a functional crosstalk between ferroptotic signaling and immune evasion. For instance, elevated expression of SLC7A11 in cervical tumors has been associated with reduced CD8^+^ T cell infiltration and resistance to immune checkpoint therapy (10). Moreover, HPV oncoproteins influence ferroptosis sensitivity via regulation of p53 and NRF2 pathways, offering a mechanistic link between viral oncogenesis and ferroptotic control (11, 12). These emerging insights highlight the dual role of ferroptosis in cervical cancer, not only as a cell death modality but also as a pivotal modulator of tumor immunity. This mini-review aims to synthesize recent advances in our understanding of ferroptosis–immune crosstalk in cervical cancer, focusing on mechanistic underpinnings and therapeutic implications, with an eye toward novel combination strategies to overcome immune resistance.

## 2 Molecular mechanisms of ferroptosis in cervical cancer

Ferroptosis is a regulated form of cell death driven by iron-dependent lipid peroxidation and impaired redox homeostasis (7–13). It is morphologically and biochemically distinct from apoptosis, necroptosis, and other cell death modes. Canonical ferroptosis involves excessive accumulation of ROS, particularly lipid ROS, resulting from disturbed iron metabolism, depletion of glutathione (GSH), and inactivation of glutathione peroxidase 4 (GPX4). Additional protective systems, including the FSP1–CoQ10 axis and the recently discovered mitochondrial DHODH–CoQ10 pathway, also counteract ferroptotic lipid peroxidation. Key regulators of ferroptosis include GPX4, SLC7A11, ACSL4, and iron metabolism proteins such as TFR1, ferritin, and ferroportin. When these defense systems are disrupted, cells become highly sensitive to ferroptotic cell death, especially under oxidative stress. In the following sections, we focus on how these mechanisms are uniquely regulated or altered in cervical cancer, particularly under the influence of HPV oncoproteins (**Table 1**).

**Table 1:** Key ferroptosis-related genes and immune modulators in cervical cancer.

Gene/molecule	Function in ferroptosis	Role in immune modulation	Alteration in cervical cancer	Therapeutic implication
GPX4 (Glutathione peroxidase 4)	Detoxifies lipid peroxides; suppresses ferroptosis	Maintains T cell viability by limiting ROS	Frequently up-regulated in cervical tumors	GPX4 inhibitors may induce ferroptosis and potentiate antitumor immunity
SLC7A11 (xCT)	Imports cystine for glutathione synthesis; prevents ferroptosis	Downregulated by IFN-γ, linking ferroptosis to T cell-mediated immunity	Upregulated in cervical cancer tissues	Inhibition sensitizes tumors to ferroptosis and enhances immunotherapy
ACSL4 (Acyl-CoA synthetase long-chain family member 4)	Facilitates PUFA incorporation into phospholipids; promotes lipid peroxidation and ferroptosis	Promotes ferroptotic cell death and influences inflammatory signaling	Upregulated in some cervical cancer models	Biomarker for ferroptosis sensitivity and therapeutic target
TFR1 (TFRC)	Mediates iron uptake, increasing labile iron pool	Enhances ferroptosis susceptibility; may influence iron-driven immune signaling	Overexpressed in HPV16-positive cervical tumors	Target for iron modulation and ferroptosis sensitization
FTH1 (Ferritin heavy chain 1)	Stores iron, reducing free iron and ferroptosis risk	Maintains iron homeostasis; modulates antigen presentation	Often downregulated in cervical cancer	Low expression correlates with increased ferroptotic sensitivity
HMOX1 (HO-1)	Degrades heme to release free iron; promotes ferroptosis under stress	Participates in DAMP release; modulates dendritic cell activation	Induced by oxidative stress and ferroptotic inducers	Targeting HO-1 may enhance ferroptotic response
NRF2 (NFE2L2)	Master regulator of antioxidant response; induces GPX4, SLC7A11	Reduces ROS-mediated immune activation	Frequently activated in advanced cervical lesions	NRF2 inhibitors may overcome resistance to ferroptosis
HMGB1 (High mobility group box 1)	Released by ferroptotic cells as a DAMP molecule	Activates dendritic cells and promotes antitumor immunity	Elevated after ferroptosis induction in tumors	Can serve as an immunogenic signal to promote immune surveillance
IFN-γ	Suppresses SLC7A11 and SLC3A2 expression, increasing ferroptosis	Activates CD8^+^ T cells; modulates tumor-immune microenvironnement	Reduced in immune-excluded tumors	Enhances efficacy of ferroptosis inducers when combined
PD-L1 (CD274)	Indirectly regulated by ROS and ferroptotic stress	Suppresses T cell function via immune checkpoint signaling	Overexpressed in cervical cancer	Ferroptosis inducers may synergize with PD-1/PD-L1 blockade
p53 (TP53)	Represses SLC7A11 transcription, promoting ferroptosis	Regulates immune escape via multiple pathways	Degraded by HPV E6 in cervical tumors	Reactivation of p53 could restore ferroptosis and immune surveillance
CNIH4 (Cornichon family AMPA receptor auxiliary protein 4)	Recent studies suggest inhibition of ferroptosis	May contribute to immunosuppressive TME remodeling	Upregulated in cervical cancer; linked to poor prognosis	Emerging target for ferroptosis-inducing strategies
ALOX15	Catalyzes lipid peroxidation, promoting ferroptosis	Modulates inflammation via lipid mediators	Expressed variably in cervical tumors	Targetable to promote ferroptosis in resistant tumors
SLC3A2	Part of the cystine/glutamate transporter system Xc−	Works with SLC7A11; regulated by IFN-γ	Expression modulated in cervical cancer	Co-targeting with SLC7A11 may enhance ferroptosis
DPP4 (Dipeptidyl peptidase 4)	Facilitates ferroptosis via lipid peroxidation	Links metabolic stress and immune activation	Altered in some cervical cancer contexts	Possible target for combination ferroptosis-immunotherapy strategies
ALOX12	Generates lipid peroxides; promotes ferroptosis in p53-positive cells	Can mediate immune response via lipid-derived mediators	Expression correlated with tumor suppressor activity	Promotes p53-dependent ferroptosis; therapeutic enhancer
SLC2A1 (GLUT1)	Facilitates glucose uptake, fuels GSH synthesis to suppress ferroptosis	Alters tumor metabolism affecting immune cell glucose competition	Identified in FRG prognostic signature onlinelibrary.wiley.comsciencedirect.com+15pmc.ncbi.nlm.nih.gov+15ncbi.nlm.nih.gov+15	Targeting GLUT1 may sensitize tumors to ferroptosis and reprogram immunity
CA9 (Carbonic anhydrase IX)	Modulates pH, promotes survival under ferroptotic stress	Regulates hypoxia-mediated immunosuppression	High expression correlates with poor prognosis in CC	CA9 inhibitors (e.g., SLC-0111) may synergize with ferroptosis induction
DUOX1 (Dual oxidase 1)	Generates H_2_O_2_ and ROS, can promote ferroptosis	ROS-mediated modulation of local immune activation	Included in CC ferroptosis prognostic model	ROS modulators may leverage DUOX1 to favor ferroptotic outcomes
HELLS (Helicase lymphoid-specific)	Epigenetic regulator; suppresses ROS accumulation, inhibits ferroptosis	Affects antigen presentation and immune microenvironment	Upregulated in CC, improves ROS detoxification	Inhibiting HELLS may derepress ferroptosis and enhance immune visibility
ALOX12B	Lipoxygenase catalyzing PUFA peroxidation, drives ferroptosis	Produces lipid mediators influencing immune signaling	Expressed in CC and included in FRG signatures	Targeting ALOX12B may potentiate ferroptosis integrity
MIOX (Myo–inositol oxygenase)	Regulates Fe^2+^ and GSH levels, promotes ferroptosis	Alters redox milieu, impacting immune cell function	Identified in CC ferroptosis-related gene model	MIOX modulation could tip redox balance to trigger ferroptosis
CDO1 (Cysteine dioxygenase type 1)	Catalyzes cysteine metabolism, lowers GSH, enhances ferroptosis	Influences cysteine/GSH availability affecting T cell activity	Included in CC ferroptosis signature	CDO1 activation may synergize with other ferroptosis inducers
MTCH1 (Mitochondrial carrier homolog 1)	Suppression leads to downregulation of GPX4, increased ROS → ferroptosis	May provoke mitochondrial ROS–mediated immune signaling	Deficiency triggers ferroptosis in CC cells	Targeting MTCH1–FoxO1–GPX4 axis offers novel combination approach
circEPSTI1 (circular RNA)	Suppresses SLC7A11 → reduces GSH → promotes ferroptosis	Alters immune microenvironment via redox signaling	Knockdown induces ferroptosis in cervical cancer	circRNAs may represent a new regulatory node for therapy
circACAP2/miR-193a-5p	circACAP2 sponges miR-193a-5p → GPX4 upregulation → inhibits ferroptosis	Modulates tumor cell death immunogenicity	High expression in CC linked to ferroptosis resistance	Targeting circACAP2/miR-193a-5p axis to trigger ferroptosis
KRAS/NRAS/HRAS	Oncogenic RAS signaling modulated during SIL-to-SCC transitions with ferroptosis	RAS pathway influences immune evasion and metabolic reprogramming	RAS expression changes correlate with lesion progression	RAS modulation may influence ferroptosis sensitivity
FANCD2, FANCA, MTOR, STMN1, RRM2, AURKA	DNA repair/cell cycle regulators that may indirectly modulate ferroptosis via oxidative stress	Affect DNA damage response and tumor antigen release	Highlighted in pan–cancer FRG analysis including CC	Combination therapies may exploit DDR inhibition + ferroptosis
PRDX1, ATG4D	Antioxidant/cytoprotective genes; regulate ROS and ferroptosis susceptibility	Autophagy–ferroptosis crosstalk influences immune cell interactions	Noted in CC FRG gene–gene co–occurrence	Targeting antioxidant defenses could amplify ferroptotic potential
FSP1, GCH1	GPX4-independent ferroptosis suppressors via CoQ/BH_4_ pathways	Influence redox balance under glutathione-depleted conditions	Implicated in broader ferroptosis but yet unstudied in CC	Novel inhibitory axes for ferroptosis–immune therapeutic synergy

### 2.1 Iron metabolism drives ferroptosis through redox dysregulation

**Figure 1:**
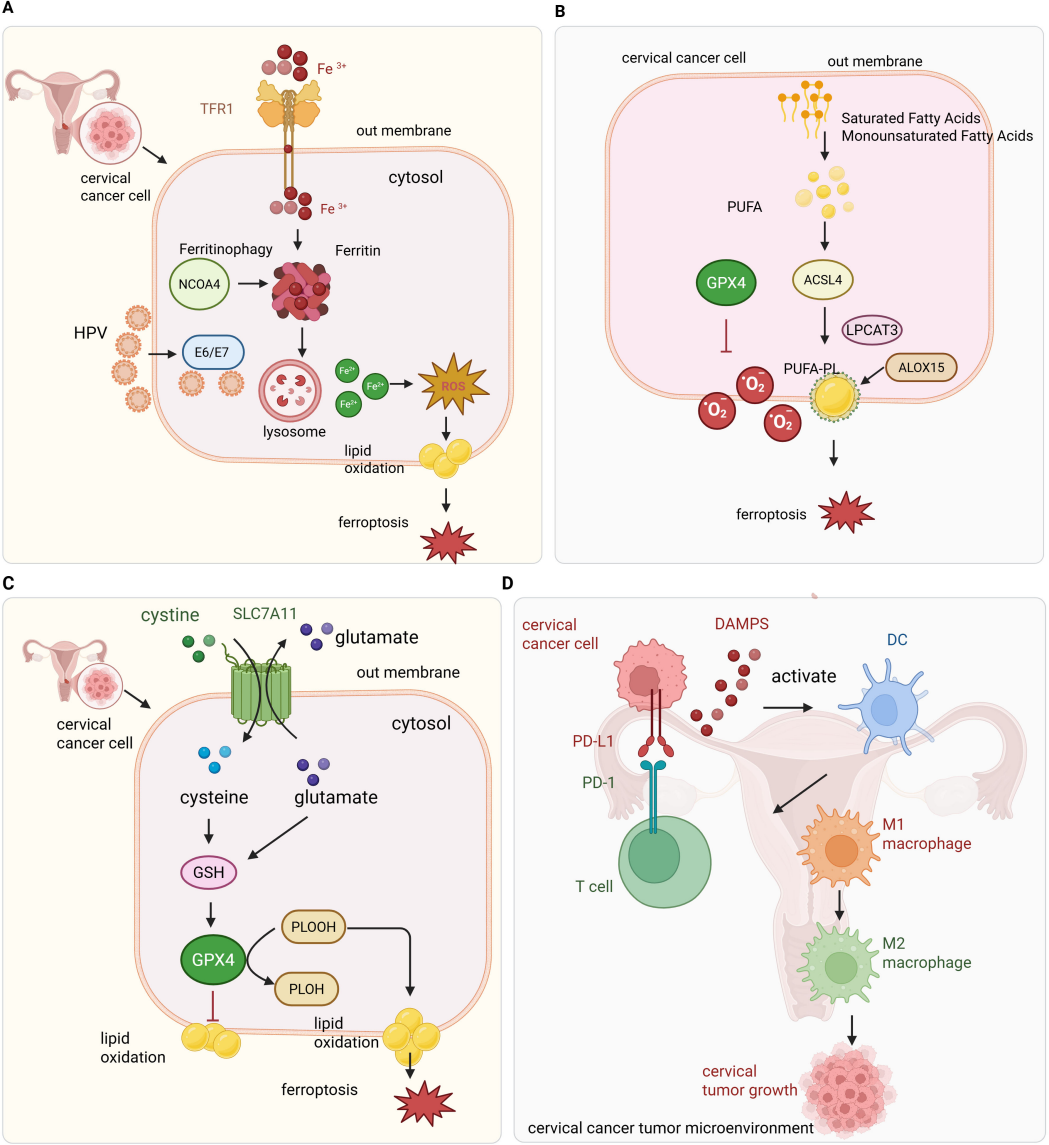
Overview of ferroptosis regulation and immune interactions in cervical cancer. **(A)** HPV oncoproteins dysregulate iron metabolism by increasing iron uptake through TFR1 and promoting ferritin degradation via NCOA4-mediated ferritinophagy, resulting in elevated Fe^2+^ and ROS production through the Fenton reaction. **(B)** Lipid metabolism enzymes such as ACSL4 facilitate incorporation of polyunsaturated fatty acids (PUFAs) into membranes, where ROS-induced lipid peroxidation triggers ferroptosis. Key antioxidant defenses including GPX4, SLC7A11, FSP1, and NRF2 mitigate lipid peroxidation to protect tumor cells. **(C)** Intracellular antioxidant defense against ferroptosis in cervical cancer cells. The cystine/glutamate antiporter SLC7A11 (system Xc^−^) imports extracellular cystine, which is reduced to cysteine inside the cell and used for glutathione (GSH) synthesis. GPX4 utilizes GSH to detoxify lipid peroxides (PLOOH) to non-toxic lipid alcohols (PLOH), preventing ferroptotic cell death. SLC7A11 exchanges intracellular glutamate for extracellular cystine, maintaining redox balance. This antioxidant system is critical for cervical cancer cell survival by suppressing lipid peroxidation and ferroptosis. **(D)** Ferroptotic tumor cells release immunogenic signals that activate immune cells; however, the oxidative microenvironment promotes immune suppression via checkpoint molecules like PD-L1, facilitating tumor immune evasion.

Ferroptosis in cervical cancer is tightly linked to altered iron metabolism, HPV-induced reprogramming of iron-regulatory genes, and enhanced ferritin degradation. Together, these factors converge to elevate intracellular iron and oxidative stress (**Figure 1A**).

#### 2.1.1 Cellular iron homeostasis and ferroptosis susceptibility

Iron metabolism is essential for cellular functions such as DNA synthesis, oxygen transport, and mitochondrial respiration (14). However, dysregulated iron homeostasis generates excessive ROS via the Fenton reaction, which forms highly reactive hydroxyl radicals (•OH) that initiate lipid peroxidation—central to ferroptosis (15). Ferroptosis is characterized by iron-dependent accumulation of lipid ROS leading to oxidative membrane damage and cell death (16).

The major contributors to intracellular iron regulation include transferrin (TF), transferrin receptor 1 (TFR1), divalent metal transporter 1 (DMT1), ferritin (FTH1 and FTL), and ferroportin (SLC40A1) (17). Under physiological conditions, transferrin binds extracellular Fe^3+^, which is internalized through TFR1-mediated endocytosis. Inside the endosome, Fe^3+^ is reduced to Fe^2+^ and released into the cytosol by DMT1, entering the labile iron pool (LIP). Ferritin stores excess iron, while ferroportin exports iron to maintain balance. Cancer cells demand high levels of iron for rapid growth, resulting in upregulated transferrin receptor 1 (TfR1) to increase iron uptake. This elevated TfR1 expression enables targeted therapeutic strategies, such as TransTACs, which exploit TfR1-mediated endocytosis to selectively degrade membrane proteins crucial for tumor survival and drug resistance, offering a novel approach for precise cancer treatment (18). Importantly, iron overload is not just a byproduct of tumor metabolism but a necessary driver of malignant transformation (19). High LIP levels enhance mitochondrial ROS production and lipid peroxidation. These redox-active conditions are particularly pronounced in rapidly proliferating cervical cancer cells, which require increased iron for nucleotide synthesis but become vulnerable to ferroptosis if antioxidant defenses are compromised.

#### 2.1.2 HPV-driven alteration of iron-regulatory genes in cervical cancer

In cervical cancer, the iron input-export pathway is disrupted, HPV infection induces changes in iron regulatory proteins, leading to altered ferroptosis. While ferroptosis occurs during precancerous squamous intraepithelial lesions (SIL), cervical squamous cell carcinoma (CSCC) cells develop anti-ferroptotic mechanisms that enable survival and oncogenesis (20). In addition, HPV oncoproteins E6 and E7 not only interfere with p53 and retinoblastoma protein (pRb) but also impact metabolic gene expression, including iron-regulatory genes (21). E6 upregulates TFR1 and DMT1, while suppressing FTH1 expression via epigenetic silencing. This promotes iron accumulation in HPV-transformed cells, establishing a redox-unstable environment conducive to ferroptosis initiation (22). Furthermore, HPV-driven inflammation and oxidative stress activates the iron storage release mechanism through ferritin degradation (23). Thus, iron becomes more available for catalyzing lipid oxidation, increasing ferroptosis vulnerability. Yet, tumor cells may simultaneously activate compensatory antioxidant pathways to evade this outcome, forming the basis for ferroptosis resistance in advanced cervical tumors.

#### 2.1.3 Ferritinophagy releases stored iron and amplifies ferroptotic potential

Ferritinophagy, the autophagic degradation of ferritin, is a selective process that liberates stored iron into the cytosol. This process is mediated by the cargo receptor NCOA4 (nuclear receptor coactivator 4), which binds ferritin and targets it to the lysosome (24, 25). Degradation of ferritin increases the intracellular Fe^2+^ pool, driving the Fenton reaction and ROS generation, thereby sensitizing cells to ferroptosis. In cervical cancer, NCOA4 promotes ferroptosis by mediating ferritinophagy, which increases the intracellular labile iron pool and reactive oxygen species (ROS) through enhanced Fenton reactions. This mechanism is activated by dihydroartemisinin (DHA), sensitizing cervical cancer cells to ferroptotic cell death and enhancing the efficacy of doxorubicin (26). Moreover, inhibition of autophagy with chloroquine abrogated this effect, confirming that ferritinophagy is necessary for this form of cell death (27). Interestingly, radiation and chemotherapeutic stress may activate NCOA4-mediated ferritinophagy in cervical tumors as a cellular attempt to recycle iron. When combined with ferroptosis inducers, this response becomes maladaptive, leading to extensive oxidative injury and cell death (28). These findings suggest that modulation of ferritinophagy could enhance the therapeutic index of ferroptosis-based interventions.

### 2.2 Lipid metabolism promotes ferroptosis through PUFA peroxidation

#### 2.2.1 Polyunsaturated phospholipid biosynthesis and lipid ROS accumulation

The defining feature of ferroptosis is the peroxidation of polyunsaturated fatty acid-containing phospholipids (PUFA-PLs) in cell membranes. These PUFA-PLs are highly susceptible to oxidative damage due to the presence of bis-allylic hydrogen atoms. The biosynthesis of PUFA-PLs involves the activation of free PUFAs by Acyl-CoA synthetase long-chain family member 4 (ACSL4) and their incorporation into membrane phospholipids via lysophosphatidylcholine acyltransferase 3 (LPCAT3) (29). ACSL4 catalyzes the esterification of PUFAs into their corresponding acyl-CoA derivatives. These activated fatty acids are then incorporated into phospholipids within cellular membranes, rendering them highly susceptible to oxidative attack during ferroptosis. ACSL4 determines the lipid composition of cell membranes by selectively enriching PUFA-phospholipids, particularly phosphatidylethanolamines, that serve as substrates for enzymatic peroxidation (**Figure 1B**). This biochemical specificity defines the ferroptosis sensitivity profile of cancer cells. Loss of ACSL4 function results in a phospholipid landscape dominated by monounsaturated species, which are comparatively inert to peroxidation and ferroptosis. ACSL4 also mediates the anticancer effects of oleanolic acid (OA) in cervical cancer by promoting ferroptosis (30). Cervical tumor cells may exploit this pathway for immune modulation or treatment resistance. For instance, cells with high PUFA content exhibit increased susceptibility to ferroptosis when Glutathione peroxidase 4 (GPX4) is inhibited (31). These observations underscore the importance of lipid metabolism in determining ferroptosis sensitivity in the tumor microenvironment.

#### 2.2.2 Role of lipoxygenases in lipid peroxidation and ferroptosis execution

While ROS from mitochondrial metabolism or the Fenton reaction can initiate lipid oxidation, lipoxygenases (ALOXs) catalyze specific peroxidation of PUFA-PLs, playing a crucial role in ferroptosis execution (32). In particular, arachidonate 15-lipoxygenase (ALOX15) is highly expressed in cervical epithelial cells and has been shown to oxidize PE-AA to 15-HpETE-PE, a key lipid peroxide triggering ferroptosis. ALOX15 knockdown conferred resistance to RSL3-induced ferroptosis, while its overexpression enhanced lipid peroxidation and cell death. Moreover, upregulation of ALOX15 has been observed in cervical intraepithelial neoplasia, suggesting its involvement in the early stages of ferroptosis priming. ALOX15 promotes ferroptosis in cervical cancer by facilitating lipid peroxidation, and its expression is suppressed by tumor-associated macrophage-derived miRNA-660-5p, which inhibits ferroptosis and contributes to tumor progression. High ALOX15 levels correlate with better prognosis, making it a potential therapeutic and prognostic target (33).

### 2.3 Key regulators of ferroptosis and their mechanistic roles

#### 2.3.1 GPX4 detoxifies lipid peroxides and suppresses ferroptosis

GPX4 serves as a central executor of ferroptosis resistance by directly catalyzing the reduction of lipid hydroperoxides (PLOOHs) to their corresponding alcohols (PLOHs), utilizing glutathione (GSH) as a cofactor (16). Unlike other GPX isoforms, GPX4 exhibits unique substrate specificity for complex membrane phospholipid peroxides, and its inactivation is both necessary and sufficient to initiate ferroptotic death. Structurally, GPX4 harbors a selenocysteine residue at its active site, which is essential for its peroxidase activity. The availability of reduced GSH and selenocysteine incorporation during translation tightly regulates its activity. Pharmacologic inhibitors such as RSL3 bind the active site and inhibit enzymatic function, leading to an unchecked accumulation of PLOOHs and subsequent ferroptotic collapse. Genetic depletion of GPX4 produces a similar phenotype, confirming its non-redundant role.

GPX4 acts as a key anti-ferroptosis enzyme in cervical cancer, and its downregulation—triggered by MTCH1 deficiency and impaired FoxO1 nuclear translocation—leads to elevated ROS and ferroptotic cell death (34). Targeting the MTCH1–FoxO1–GPX4 axis sensitizes cervical cancer cells to ferroptosis, offering a promising therapeutic strategy. GPX4 also protects cells from ferroptosis through the NRF2/GPX4/xCT antioxidant pathway. Triptolide induces ferroptosis in cervical cancer cells by downregulating NRF2, which leads to decreased GPX4 and xCT expression, resulting in increased lipid peroxidation and tumor growth inhibition (35). In cervical cancer, elevated GPX4 expression correlates with chemoradiotherapy resistance, particularly in HPV-positive tumors. Tumor cells exhibiting mesenchymal or hypoxic signatures demonstrate GPX4 dependency for survival. Inhibition of GPX4 in these contexts induces catastrophic lipid peroxidation and irreversible mitochondrial damage. These observations highlight the potential for GPX4-targeted strategies to overcome intrinsic resistance mechanisms in cervical malignancies. Moreover, metabolic stressors such as cystine deprivation, GSH depletion, or oxidative phosphorylation inhibitors synergistically impair GPX4 function, offering avenues for therapeutic combination approaches. The essentiality of GPX4 in protecting cancer cells from ferroptotic damage underscores its centrality as a druggable target.

#### 2.3.2 SLC7A11 sustains GSH synthesis and inhibits ferroptosis

SLC7A11 encodes the light chain subunit of the cystine/glutamate antiporter system Xc^−^, which imports extracellular cystine in exchange for intracellular glutamate (**Figure 1C**). Cystine is rapidly reduced to cysteine intracellularly and serves as a rate-limiting substrate for GSH biosynthesis. Through this mechanism, SLC7A11 indirectly maintains GPX4 activity and redox equilibrium, thereby suppressing ferroptotic responses (36). Transcriptional regulation of SLC7A11 is mediated by stress-responsive transcription factors including NRF2 and ATF4. Under oxidative or metabolic stress, these factors promote SLC7A11 expression, increasing cystine uptake and buffering intracellular ROS. Conversely, tumor suppressor p53 represses SLC7A11 transcription under certain conditions, lowering cysteine levels and sensitizing cells to ferroptosis.

SLC7A11 plays a critical role in suppressing ferroptosis in cervical cancer by mediating cystine uptake for glutathione synthesis. The RACK1/miR-1275/FUT8 axis stabilizes SLC7A11 through core-fucosylation, preventing its degradation and thus inhibiting ferroptosis, which promotes cervical cancer cell survival and progression (37). In addition, in cervical cancer, fatty acid synthase (FASN) promotes cisplatin resistance by upregulating SLC7A11, which suppresses ferroptosis. Inhibition of FASN reduces SLC7A11 expression, thereby enhancing ferroptosis and restoring cisplatin sensitivity, suggesting that targeting the FASN/SLC7A11 axis may overcome chemotherapy resistance (38). Under hypoxia-like conditions, cervical cancer cells increase SLC7A11 expression through KDM4A SUMOylation at the K471 site, which reduces H3K9me3-mediated repression of SLC7A11, thereby enhancing GPX4 levels and promoting ferroptosis resistance (39). SLC7A11 promotes cervical cancer cell survival by mediating cystine uptake to maintain glutathione synthesis, thereby inhibiting ferroptosis (40). Targeting SLC7A11 with specific inhibitors disrupts redox balance, increases ROS, and induces ferroptotic cell death, representing a promising therapeutic strategy for cervical cancer. This mechanism contributes to the survival of cervical cancer cells by inhibiting ferroptotic cell death. Targeting SLC7A11 may potentiate ferroptosis-based therapeutic strategies and disrupt redox adaptation in treatment-resistant tumors.

#### 2.3.3 FSP1 reduces CoQ10 and suppresses ferroptosis independently of GPX4

Ferroptosis suppressor protein 1 (FSP1), previously known as AIFM2, functions as an NAD(P)H-dependent oxidoreductase that reduces ubiquinone (CoQ10) to ubiquinol (CoQ10H_2_), a lipophilic radical-trapping antioxidant (41). This reaction occurs at the plasma membrane and is independent of the canonical GPX4-GSH axis, representing a parallel defense system against ferroptotic lipid damage. FSP1 is myristoylated at the N-terminus, allowing for membrane localization where lipid peroxidation is initiated. Upon CoQ10 reduction, ubiquinol neutralizes lipid radicals and halts the propagation of peroxidation chains, thus preventing membrane rupture and ferroptotic death. Cells with intact FSP1 can survive in the absence of GPX4 activity if sufficient CoQ10 and NAD(P)H are available.

In cervical cancer, treatments like propofol and paclitaxel can synergistically induce ferroptosis by modulating the ubiquinol/CoQ10/FSP1 pathway, promoting oxidative stress and ferroptotic mitochondrial damage, which enhances cancer cell death beyond apoptosis (42). In cervical cancer, high FSP1 expression has been observed in resistant tumor clones and is associated with recurrence after chemoradiotherapy. Genetic knockdown or pharmacologic inhibition of FSP1 enhances the efficacy of GPX4 inhibitors, suggesting that dual blockade of ferroptosis defense pathways can overcome therapeutic resistance. Furthermore, CoQ10 biosynthesis is tightly regulated by the mevalonate pathway, which is frequently upregulated in tumors (43). Statins or mevalonate pathway inhibitors may reduce CoQ10 availability and sensitize cervical cancer cells to ferroptosis through indirect impairment of the FSP1 pathway (44). These interactions highlight the potential of FSP1 as a therapeutic vulnerability in ferroptosis-based interventions.

#### 2.3.4 The NRF2 signaling in cervical cancer and ferroptosis regulation

NRF2 (NFE2L2) is a master transcriptional regulator of antioxidant and detoxification pathways, safeguarding cells from oxidative stress (45). When Keap1 fails to sequester NRF2, NRF2 accumulates in the nucleus, reducing cancer cell sensitivity to ferroptosis [88]. Tossetta et al. demonstrated that the NRF2/Keap1 axis is critically involved in cervical and endometrial carcinogenesis by modulating gene expression profiles that contribute to chemotherapy resistance. Inhibiting inducible NRF2 may synergize with ferroptosis-inducing strategies in cancer therapy. Additionally, NRF2 activity is modulated by its interaction with prolyl isomerase PIN1 (46). In cervical cancer, HELLS promotes tumor cell proliferation by repressing NRF2 expression, suggesting that targeting HELLS could restore ferroptosis sensitivity (47). Another downstream effector, heme oxygenase–1 (HO–1), exhibits context-dependent roles in cervical cancer: while the NRF2/HO–1 pathway generally suppresses ferroptosis due to its antioxidative function, HO–1 can switch to a pro-ferroptotic role under certain stress conditions (48). For example, CENPF knockdown leads to decreased NRF2 activity and HO–1 expression, weakening the cellular antioxidant defense (49). Conversely, Erastin treatment increases ROS and upregulates both NRF2 and HO–1 in HeLa cells; knocking down HO–1 reduces Erastin’s inhibition of colony formation, migration, invasion, and ROS generation, implying HO–1’s facilitative role in ferroptosis-mediated anticancer effects (50). The dualistic nature of HO–1 highlights its importance in cervical cancer progression and therapy response. Moreover, the Wnt signaling pathway, which is frequently dysregulated in various cancers, can modulate NRF2 expression, identifying Wnt–NRF2 crosstalk as a promising target for ferroptosis-centric therapies in cervical cancer (51).

Besides all mentioned above, several additional regulators have been implicated in ferroptosis control. For example, mitochondrial enzyme DHODH was shown to suppress ferroptosis via an alternative CoQ10-dependent mechanism in mitochondria, offering protection when GPX4 or FSP1 is compromised (52). DHODH is upregulated in cervical cancer and acts as a ferroptosis defender (53); its inhibition promotes ferroptosis and suppresses tumor cell proliferation. Combined DHODH inhibition and cisplatin synergistically enhance ferroptosis by downregulating the mTOR pathway, offering a potential therapeutic strategy for cervical cancer (53). In addition, Cornichon family AMPA receptor auxiliary protein 4 (CNIH4) is upregulated in cervical cancer, CHIH4 promotes tumor progression by inhibiting ferroptosis by enhancing SLC7A11-mediated cystine import, boosting glutathione synthesis and GPX4 activity (54). Silencing CNIH4 or SLC7A11 restores ferroptotic sensitivity, identifying CNIH4 as a potential prognostic biomarker and therapeutic target in cervical cancer. Together, these emerging regulators illustrate the complexity of ferroptosis networks and highlight the potential for multi-targeted interventions in ferroptosis modulation.

## 3 Ferroptosis and immune regulation in cervical cancer

### 3.1 Ferroptosis disrupts antigen presentation and impairs immune recognition

Effective immune surveillance relies on tumor antigen presentation via major histocompatibility complex class I (MHC-I) molecules and subsequent recognition by cytotoxic T lymphocytes (55, 56). However, many cervical cancer cells, particularly those influenced by HPV oncogenes, downregulate MHC-I expression, impairing T cell-mediated cytotoxicity (57, 58). Recent studies have demonstrated that ferroptosis contributes to this immune escape process by modulating key components of antigen processing and presentation. Ferroptotic cancer cells impair dendritic cell maturation and antigen cross-presentation, thereby suppressing adaptive immune responses and weakening antitumor immunity (59).

Lipid peroxidation, the central biochemical hallmark of ferroptosis, has been shown to disrupt endoplasmic reticulum (ER) homeostasis and interfere with the biosynthesis and trafficking of MHC-I molecules (60, 61). Oxidized phospholipids impair peptide loading onto MHC-I, leading to suboptimal antigen presentation. In HPV-transformed cervical cancer cells, where oxidative stress and redox imbalance are already prominent, induction of ferroptosis further compromises antigen processing machinery, reinforcing immune escape. Furthermore, iron accumulation within ferroptosis-prone tumor cells generates ROS, which suppress proteasome activity and alter peptide repertoire generation. Reduced proteasomal degradation of viral and neoantigenic peptides weakens the formation of immunogenic MHC-I complexes (62). However, ferroptosis-related lipid peroxidation in post-synaptic dendritic cells (psDCs) enhances MHC-I expression and facilitates their licensing, thereby promoting effective CD8^+^ T cell activation and adaptive immunity (63).

### 3.2 Ferroptosis-derived DAMPs shape the immune microenvironment

Ferroptosis can release DAMPs that activate immune responses. However, in cervical cancer, this process tends to favor immunosuppressive remodeling rather than stimulate effective anti-tumor immunity (**Figure 1D**). This is largely due to the oxidative stress and HPV-induced immunomodulation that shift the TME toward immune tolerance. During ferroptosis, DAMPs such as high mobility group box 1 (HMGB1), ATP, and oxidized phospholipids are released into the extracellular space (64). These signals are typically recognized by pattern recognition receptors (PRRs) on dendritic cells and macrophages, initiating inflammatory signaling cascades. In cervical cancer, however, prolonged exposure to oxidized lipids promotes the differentiation of tolerogenic dendritic cells and the recruitment of regulatory T cells (Tregs), which suppress cytotoxic immune responses (65). In addition, ferroptosis-induced ROS stimulate the secretion of immunosuppressive cytokines, such as interleukin-10 (IL-10) and transforming growth factor-beta (TGF-β), further inhibiting effector T cell activity. Tumor-associated macrophages (TAMs), which are abundant in cervical cancer lesions, are skewed toward an M2-like phenotype under the influence of lipid-derived DAMPs. This polarization contributes to angiogenesis, extracellular matrix remodeling, and suppression of anti-tumor immunity (66). Thus, ferroptosis can paradoxically support immune evasion through inflammation-induced immune suppression.

### 3.3 Ferroptosis reshapes immune cell infiltration in the tumor microenvironment

The tumor immune microenvironment in cervical cancer is highly heterogeneous and shaped by multiple factors, including viral oncogenes, cytokine networks, and metabolic states (67, 68). Ferroptosis represents an additional layer of immune modulation, altering the composition and function of infiltrating immune cells. Ferroptosis inducers modify chemokine expression profiles in tumor cells, leading to increased recruitment of CD8^+^ T cells and natural killer (NK) cells. In models of ferroptosis-sensitive tumors, lipid peroxidation products enhance CXCL10 and CCL5 secretion, favoring cytotoxic immune infiltration. However, in cervical cancer, the effectiveness of this recruitment is often limited by concurrent immunosuppressive signals. In contrast, ferroptosis-induced inflammation may also recruit myeloid-derived suppressor cells (MDSCs) and Tregs, depending on the balance of DAMPs and cytokines released. For instance, 4-hydroxynonenal (4-HNE), a major lipid peroxidation product, induces prostaglandin E2 (PGE2) synthesis in TAMs, which supports MDSC expansion and inhibits dendritic cell maturation (69). In addition, immunotherapy-activated CD8^+^ T cells promote tumor ferroptosis by releasing IFN-γ, which suppresses SLC3A2 and SLC7A11 expression, reduces cystine uptake, and increases lipid peroxidation—thereby enhancing antitumor efficacy and suggesting ferroptosis as a synergistic mechanism with checkpoint blockade therapy (70).

### 3.4 Ferroptosis interacts with immune checkpoint pathways

Immune checkpoint inhibitors (ICIs), including PD–1/PD–L1 and CTLA–4 antagonists, have transformed cancer treatment by reinvigorating exhausted CD8^+^ T cells. However, many patients with cervical cancer still exhibit poor responses due to a “cold” TME and insufficient T–cell infiltration (71). Ferroptosis induction synergizes with ICIs by enhancing tumor immunogenicity and overcoming immune resistance. In multiple tumor models, combining GPX4 inhibitors with anti–PD-1 therapy improved tumor responses and increased activated CD8^+^ T cell and NK cell infiltration. Specifically, IFN–γ released from ICI–activated CD8^+^ T cells downregulates system xc– components (SLC3A2 and SLC7A11) in tumor cells, thereby promoting lipid peroxidation and ferroptosis (72). Critically, PD–1 pathway blockade also protects immune effector cells from ferroptosis. A recent study showed that dendritic cells (DCs) expressing PD–L1 are protected from ferroptotic cell death during chemotherapy; PD–L1 maintains SLC7A11 expression in DCs, preventing membrane lipid peroxidation and preserving T–cell priming capacity (73). This suggests dual roles for the PD–1/PD–L1 axis: promoting ferroptosis in tumor cells while safeguarding APCs.

HPV-driven modulation of ferroptosis pathways distinctly impacts ferroptosis-immune interactions compared to non-HPV cancers. HPV oncoproteins such as E6 and E7 suppress key ferroptosis regulators including ACSL4 and ALOX15, limiting lipid peroxidation and ferroptotic cell death, thereby facilitating immune escape and contributing to resistance to ICIs (33). Additionally, HPV-induced metabolic reprogramming and oxidative stress alter the tumor immune microenvironment by affecting immune cell susceptibility to ferroptosis, further skewing immune responses toward tolerance. These HPV-specific mechanisms underscore the challenges and opportunities for combining ferroptosis induction with immunotherapy in cervical cancer. High PD–L1 expression in cervical tumors correlates with poor prognosis and immune suppression. Preclinical data from other cancers hint that combining PD–1 blockade with ferroptosis inducers may reprogram TAMs and DCs toward pro-immunity, potentially overcoming HPV-mediated tolerance. Pembrolizumab, a PD–1 inhibitor, is FDA-approved for advanced cervical cancer, combining it with ferroptosis inducers may reprogram TAMs and DCs toward immune activation. Though clinical data in cervical cancer is still limited, preclinical studies support the rationale for combined immuno-ferroptotic therapy.

### 3.5 Ferroptosis of immune cells and its implications in cervical cancer

Ferroptosis is not limited to tumor cells—immune cells themselves can undergo ferroptotic death, which can either limit or facilitate anti-tumor immunity depending on context (74). CD8^+^ T cells and NK cells, particularly under oxidative stress or nutrient deprivation in the tumor microenvironment, are vulnerable to ferroptosis due to high metabolic demands and limited antioxidant capacity (8–75). Depletion of GPX4 or cystine transport leads to dysfunctional T cell responses, impaired cytokine secretion, and eventual cell death. In cervical cancer, HPV-induced metabolic remodeling and hypoxia may further predispose infiltrating lymphocytes to ferroptosis, weakening the immune response. Dendritic cells (DCs) also rely on SLC7A11 and GPX4 to maintain their function and survival during antigen presentation. Loss of ferroptosis protection in DCs impairs T cell priming, tipping the balance toward immune evasion (65). However, controlled ferroptosis in immunosuppressive cells may offer a therapeutic opportunity to remodel the TME toward immunogenicity. Thus, understanding the ferroptosis susceptibility of different immune cell subsets is essential for designing combination therapies that protect beneficial immune cells while sensitizing tumor and suppressive immune populations.

## 4 Therapeutic potential and future perspectives

### 4.1 Ferroptosis inducers offer a novel strategy for cervical cancer treatment

The pharmacologic induction of ferroptosis has emerged as a promising therapeutic strategy in malignancies characterized by resistance to conventional treatments. In cervical cancer, which often exhibits immune evasion and recurrence following chemoradiotherapy, ferroptosis induction represents a novel modality to overcome these therapeutic limitations.

Several ferroptosis inducers, including erastin, RSL3, and FIN56, target distinct molecular nodes such as system Xc-, GPX4, or CoQ10 pathways to induce lethal lipid peroxidation. Erastin, by inhibiting SLC7A11, depletes intracellular cystine and reduces glutathione synthesis, indirectly disabling GPX4 activity. RSL3 directly binds and inactivates GPX4, leading to accumulation of lipid peroxides. FIN56 promotes degradation of GPX4 and depletes CoQ10, disrupting antioxidant defense systems. In cervical cancer models, these agents have shown selective cytotoxicity in HPV-transformed cells, particularly those with aberrant redox metabolism and elevated iron pools. Such metabolic vulnerabilities sensitize cervical cancer cells to ferroptosis induction, highlighting a potential therapeutic window. Importantly, recent studies have demonstrated that ferroptosis inducers can sensitize cervical cancer cells to traditional treatments. For example, combining erastin with cisplatin enhances DNA damage and oxidative stress, leading to synergistic tumor cell death. Similarly, ionizing radiation upregulates ACSL4 and increases PUFA-phospholipid content, priming tumor cells for ferroptosis upon GPX4 inhibition (76). These synergistic effects support the rationale for integrating ferroptosis inducers into existing therapeutic regimens.

### 4.2 Challenges limiting the clinical translation of ferroptosis-based therapies

Despite the therapeutic promise, several challenges hinder the clinical translation of ferroptosis inducers in cervical cancer. Tumor heterogeneity, both genetic and metabolic, results in variable ferroptosis sensitivity (77, 78). Subpopulations of cervical cancer cells may harbor compensatory antioxidant pathways (e.g., FSP1, DHODH) that confer resistance to GPX4 inhibition. Therefore, patient stratification based on ferroptosis regulator expression profiles may be necessary. Another limitation involves the delivery and specificity of ferroptosis inducers, many existing compounds exhibit poor solubility, off-target toxicity, or lack tumor selectivity. Moreover, novel delivery systems, such as lipid nanoparticles or tumor-targeted prodrugs, are being explored to enhance drug bioavailability and reduce systemic toxicity. In cervical cancer, leveraging HPV-specific antigens or receptors may provide a basis for targeted delivery. Furthermore, ferroptosis inducers may activate systemic inflammation or trigger unintended ferroptosis in normal tissues, particularly in organs with high PUFA content such as the liver or brain.

### 4.3 Clinical perspectives and future directions

Although no ferroptosis-based therapy has yet received regulatory approval, multiple ferroptosis inducers are in early-phase clinical trials for solid tumors, including agents targeting GPX4, system Xc-, and iron metabolism. The development of companion diagnostics based on ACSL4, SLC7A11, or lipid peroxidation biomarkers may aid in identifying responsive patient subsets. In cervical cancer, future clinical studies should explore rational combinations of ferroptosis inducers with radiotherapy, chemotherapy, and immunotherapy. Personalized approaches integrating multi-omic profiling, including transcriptomics, lipidomics, and redox metabolomics, may optimize therapeutic strategies and predict ferroptosis susceptibility. Additionally, the temporal dynamics of ferroptosis induction—whether pulsed or sustained—may influence therapeutic outcomes and should be systematically evaluated.

## 5 Conclusion

Ferroptosis has emerged as a pivotal process influencing tumor biology and immune dynamics in cervical cancer. Here, we highlight how ferroptosis intersects with immune evasion mechanisms to shape tumor progression and treatment resistance. Specifically,

Dysregulated iron metabolism, aberrant lipid peroxidation, and impaired antioxidant defenses—shaped in part by HPV oncoproteins—modulate ferroptotic vulnerability and immune escape in cervical tumors. Key regulators such as GPX4, SLC7A11, ACSL4, and FSP1 define the ferroptosis threshold, while HPV-driven suppression of ACSL4 and ALOX15 contributes to resistance. The immune consequences of ferroptosis are context-dependent. Ferroptotic tumor cells can enhance antigenicity and support CD8^+^ T cell responses, yet also release DAMPs and ROS that promote immunosuppressive inflammation and recruitment of Tregs and MDSCs. Moreover, immune cells themselves, including T cells and dendritic cells, are vulnerable to ferroptosis, particularly in the oxidative and HPV-modulated tumor microenvironment.

Therapeutically, ferroptosis induction offers a novel strategy to combat cervical cancers that are refractory to conventional therapies. Combination approaches—such as ferroptosis inducers with immune checkpoint inhibitors—may restore anti-tumor immunity, reprogram suppressive myeloid cells, and overcome resistance to PD-1/PD-L1 blockade. Erastin, RSL3, and other small molecules have shown preclinical efficacy, particularly in HPV-transformed cells with elevated iron load and redox imbalance. Radiation and cisplatin may also sensitize tumors to ferroptosis through lipid remodeling.

In summary, ferroptosis represents both a vulnerability and a regulatory node in cervical cancer. Targeting ferroptosis—especially in combination with immunotherapy—may reshape the immune microenvironment and improve treatment outcomes. Future research should prioritize HPV-specific ferroptosis-immune interactions, immune cell protection strategies, and biomarker-driven patient selection to facilitate clinical translation.
